# Subclassification, survival prediction and drug target analyses of chemotherapy-naïve muscle-invasive bladder cancer with a molecular screening

**DOI:** 10.18632/oncotarget.25407

**Published:** 2018-05-25

**Authors:** Sebastien Rinaldetti, Eugen Rempel, Thomas Stefan Worst, Markus Eckstein, Annette Steidler, Cleo Aaron Weiss, Christian Bolenz, Arndt Hartmann, Philipp Erben

**Affiliations:** ^1^ Department of Hematology and Oncology, Medical Faculty Mannheim, University of Heidelberg, 68167 Mannheim, Germany; ^2^ German Cancer Research Center (DKFZ), Division of Signalling and Functional Genomics, 69120 Heidelberg, Germany; ^3^ Department of Stem Cell Biology, Centre of Organismal Studies, University Heidelberg, 69120 Heidelberg, Germany; ^4^ Department of Urology, Medical Faculty Mannheim, University of Heidelberg, 68167 Mannheim, Germany; ^5^ Institute of Pathology, University Erlangen-Nuremberg, 91054 Erlangen, Germany; ^6^ Institute of Pathology, University Medical Centre Mannheim, 68167 Mannheim, Germany; ^7^ Department of Urology, University of Ulm, 89075 Ulm, Germany

**Keywords:** bladder cancer subtypes, survival prediction, drug targets, gene expression, biomarkers

## Abstract

**Background:**

Transcriptome expression studies identified distinct muscle invasive bladder cancer (MIBC) subtypes closely related with breast cancer subclasses. Here we developed a sensitive quantification method for MIBC subclassification (luminal, basal, p53-like). In addition, the subtype specific expression of drug targets has been investigated.

**Methods:**

Absolute quantification (nCounter) of a 64-gene panel was performed on MIBC patients (n=47) treated exclusively with radical cystectomy (RC). In conjunction of 170 MIBCs from 3 independent cohorts, a minimal set of consensus genes has been established. Survival of the consensus subtypes has been assessed by multivariate analysis. Relevant drug targets were tested for their subtype specificity in a clustering independent assessment.

**Results:**

A reduced 36-gene panel stably clustered into 3 subtypes throughout the cohorts (luminal, basal, infiltrated). Patients treated by RC only, showed worst 8-year disease specific survival (DSS) for the luminal subtype in contrast to the infiltrated subtype (17% vs. 73%, p=0.011). In multivariate analyses, the risk stratification based on luminal versus not-luminal MIBC proved to be an independent predictor for DSS superior to the TNM system in patients with RC. Drug targets (e.g. *ERBB2, FGFR, AR, PDGFRB*) showed a distinct subtype attribution. The subtypes based on this nCounter screening could further be validated by the TCGA cohort.

**Conclusion:**

This MIBC subtype screening predicted survival and allowed an analysis of subtype specific drug targets, thus being a powerful tool for the translation of personalized MIBC treatment concepts.

## INTRODUCTION

Even though bladder cancer accounts to the top ten most common malignancies, personalized therapy concepts have not yet found their advent into clinical routine. Indeed, no targeted frontline therapies are established besides the recent approval of PD-1/PD-L1 inhibitors as a 2^nd^-line treatment against metastatic MIBC [1]. Patients with pT3-pT4 pN+ muscle invasive bladder cancer (MIBC) have a 5-year overall survival (OS) rate of only 35% [2]. MIBC accounts for 20-40% of tumor incidence [3]. The progression rates of MIBC to adjuvant chemotherapy are 50-70% [4]. Many clinical studies try to reevaluate the role of perioperative therapy concepts but are confronted to two major problems: clinical understaging and uncertain pathologic assessments especially after transurethral resection of the bladder (TURB) [5].

Transcriptome expression studies showed that the molecular phenotype of bladder cancer is highly heterogeneous. Subtypes showed typical expression profiles for basal and luminal markers comparable to the breast cancer subtypes. Furthermore, these subclasses correlated with survival and are suspected to differentially express drug targets [6–11]. Recent attempts to translate MIBC subclassification into clinics were based on NGS and microarray data [10, 12, 13]. Consensus meetings and reviews agree that further validation of the bladder cancer subtypes is needed and that a cost effective, sensitive method is required in order to translate this molecular screening into clinical routine. The subtype-specific expression of drug targets may be a powerful tool for advancing personalized therapy concepts in neoadjuvant (NAC) or adjuvant chemotherapy (AC) settings [14–17].

The scope of this study is to develop an nCounter screening for the molecular characterization of MIBC. The main translational benefit of such a screening should be a risk stratification and identification of relevant subtype specific drug targets relevant for targeted personalized bladder cancer treatment concepts.

## RESULTS

### Clinicopathologic characteristics

Clinicopathologic characteristics of the Mannheim cohort (n=47) are summarized in Table 1. Clinicopathologic characteristics of the Chungbuk and MDA cohort are summarized in Supplementary Table 1 and Supplementary Table 2.

**Table 1:** Clinicopathologic characteristics of the Mannheim cohort

Cohort characteristics	Total	(%)	Luminal	(%)	Basal	(%)	Infiltrated	(%)	p-value
Cohort size	47		9	(19)	13	(28)	25	(53)	
Median age	67		67		70		65		0.358
Female	13	(28)	3	(33)	3	(23)	7	(28)	0.912
Male	34	(72)	6	(67)	10	(77)	18	(72)	
**TNM Stage**									
pTa, pT1, pTis	3	(6)	0	(0)	0	(0)	3	(12)	0.883
pT2	11	(23)	2	(22)	3	(23)	6	(24)	
pT3	26	(55)	6	(67)	7	(54)	13	(52)	
pT4	7	(15)	1	(11)	3	(23)	3	(12)	
pN+	17	(36)	6	(67)	3	(23)	8	(32)	0.097
cM+	8	(17)	1	(11)	3	(23)	4	(16)	0.768
**Additional Therapy**									
NAC	1	(2)	0	(0)	1	(8)	0	(0)	0.467
AC	7	(16)	3	(33)	1	(8)	3	(13)	0.303

AC = adjuvant chemotherapy, NAC = neoadjuvant chemotherapy.

### Bladder cancer subtype validation

**Figure 1:**
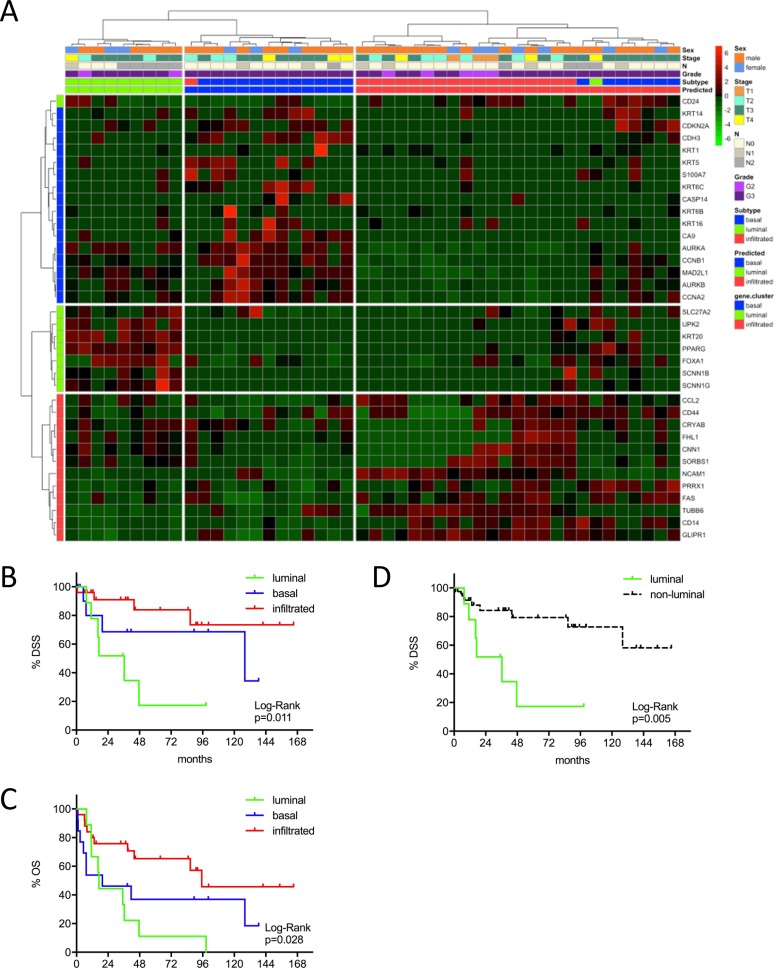
**(A)** MIBC subtype classification of the Mannheim cohort (n=47) by gene expression profiling of the reduced consensus geneset with the NanoString nCounter technology. **(B, C)** Kaplan-Meier plots of overall survival (OS) and disease specific survival (DSS) of the basal, luminal and infiltrated subtype. **(D)** Kaplan-Meier plots of DSS comparing survival of luminal versus non-luminal MIBC.

**Figure 2:**
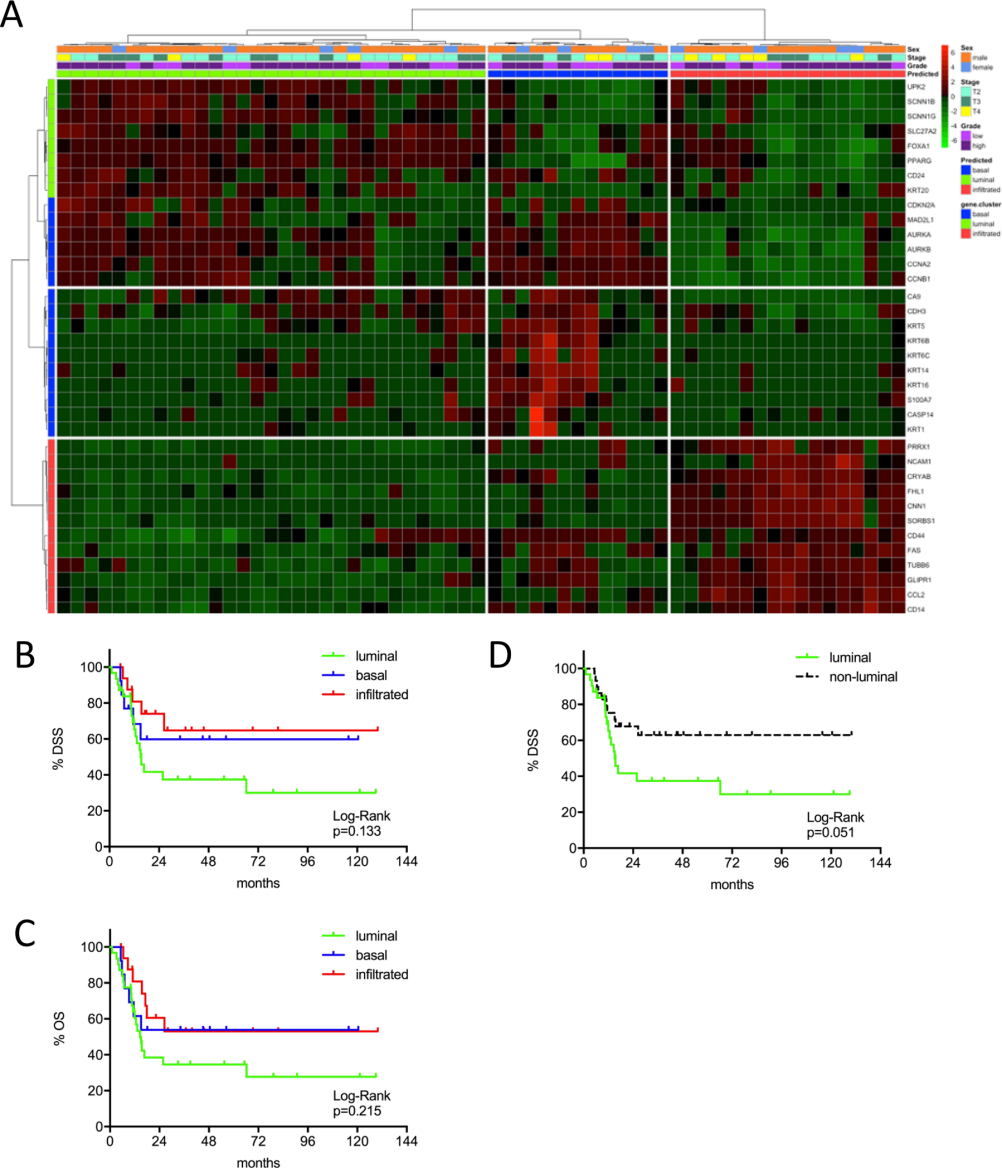
**(A)** MIBC subtype classification of the Chungbuk cohort (n=61) by gene expression profiling of the reduced consensus geneset based on in silico microarray data (GSE13507). **(B, C)** Kaplan-Meier plots of overall survival (OS) and disease specific survival (DSS) of the basal, luminal and infiltrated subtype. **(D)** Kaplan-Meier plots of DSS comparing survival of luminal versus non-luminal MIBC.

**Figure 3:**
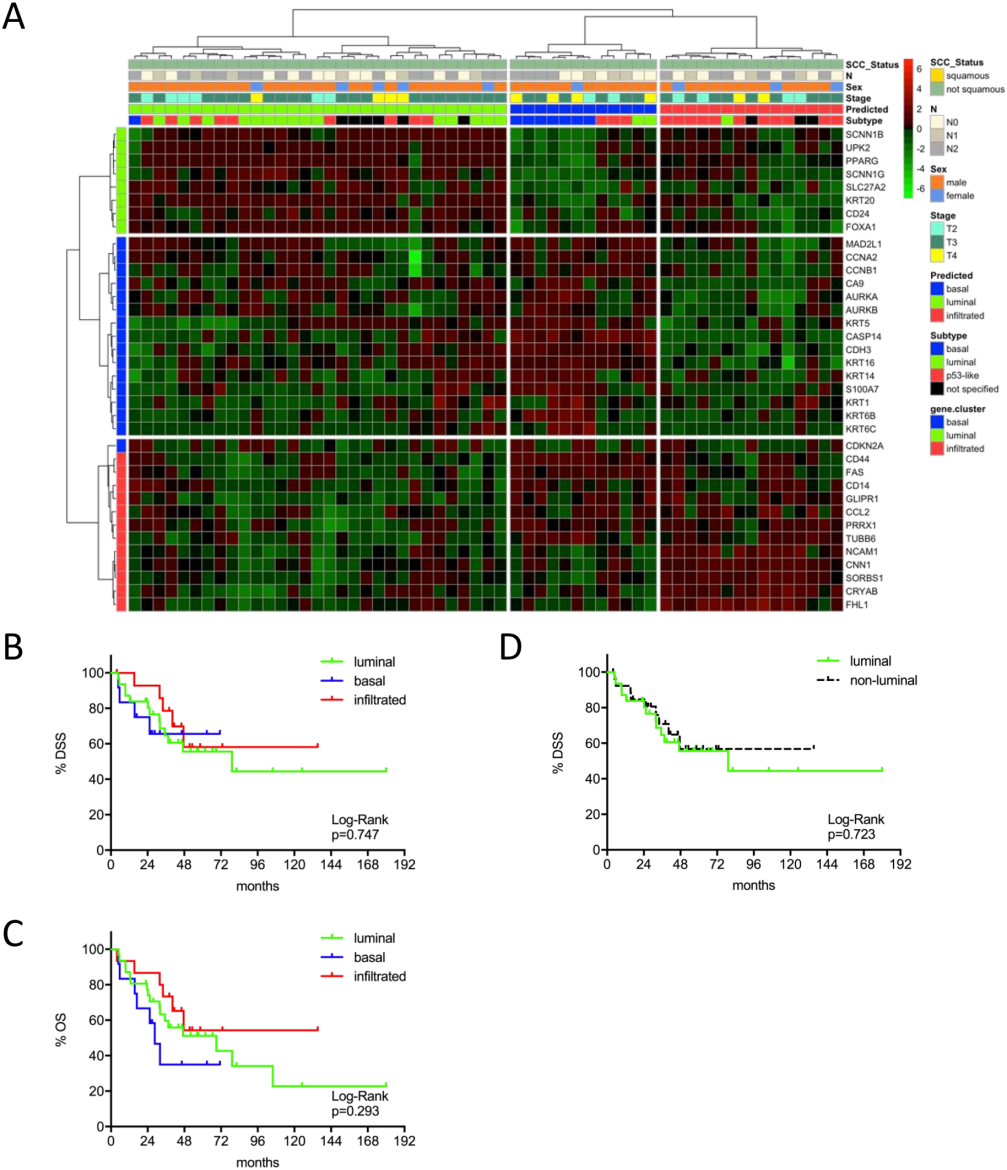
**(A)** MIBC subtype classification of the MDA cohort (n=58) by gene expression profiling of the reduced consensus geneset based on in silico microarray data (GSE48276). **(B, C)** Kaplan-Meier plots of overall survival (OS) and disease specific survival (DSS) of the basal, luminal and infiltrated subtype. **(D)** Kaplan-Meier plots of DSS comparing survival of luminal versus non-luminal MIBC.

**Figure 4:**
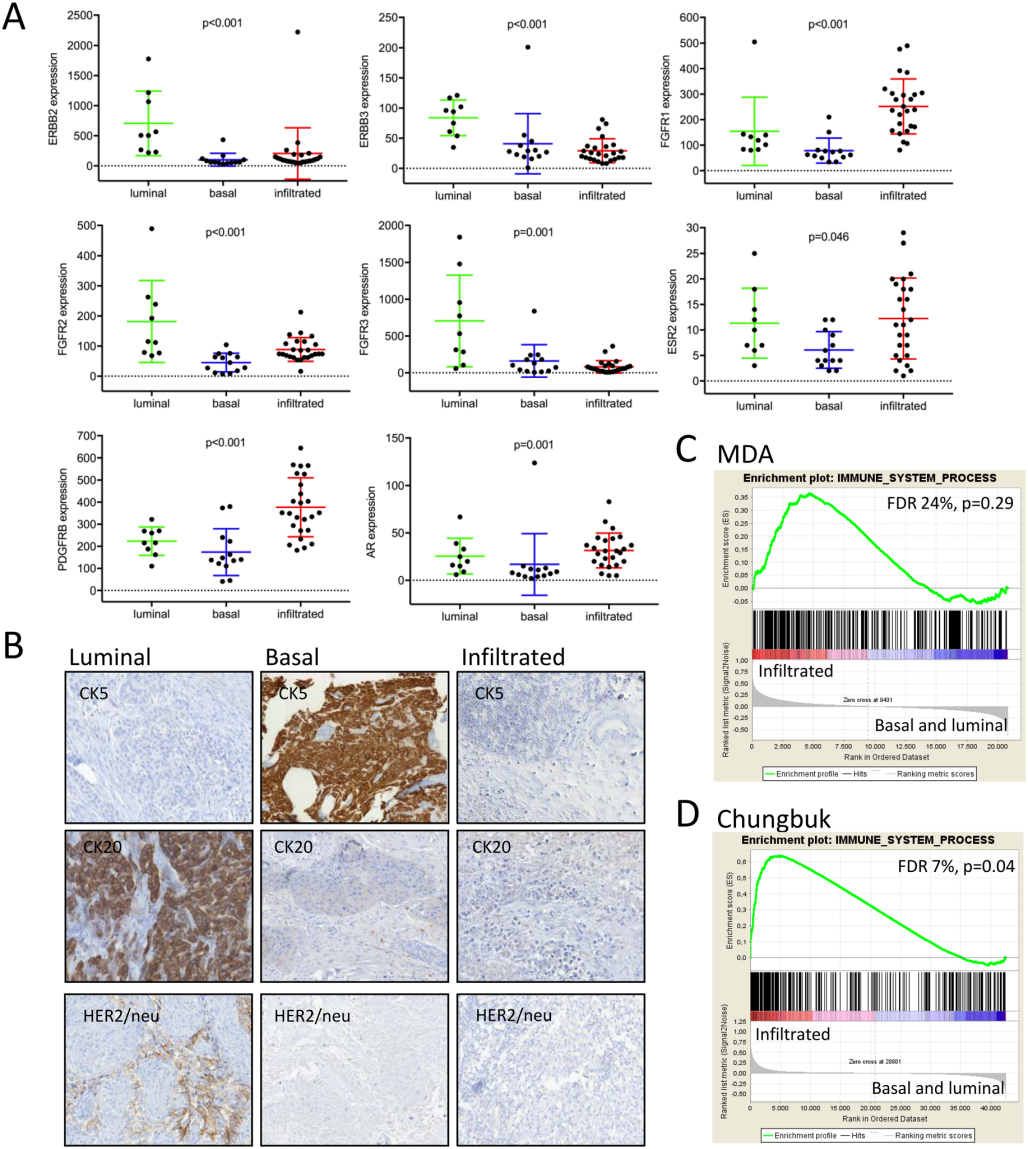
**(A)** Drug targets were tested for their subtype specific expression by the Kruskal-Wallis test. Absolute quantification of transcript levels was based on normalized nCounter counts. **(B)** Immunohistochemistry of representative luminal, infiltrated and basal MIBC. **(C, D)** Gene set enrichment analyses of an immune signature from the Gene Ontology database.

The molecular subclassification based on basal, luminal and p53-like gene signatures resulted in a consensus gene panel of 36 stable clustering genes (basal: n=16, luminal: n=8, p53-like n=12) for the Mannheim, Chungbuk, MDA and Lund cohort (Venn diagrams, Supplementary Figure 2). Nanostring nCounter technology-based expression data classified the MIBCs of the Mannheim cohort into three distinct subtypes (Figure 1). Immunohistochemistry of representative FFPE samples underlined their subtype specificity and confirmed a differential expression on protein level (Figure 4B). The subtype clusters were reproduced and validated in silico by the Chungbuk cohort (Figure 2A) and our predicted subtypes matched accurately with the original subtypes from the MDA cohort (color bars, Figure 3A and Supplementary Figure 4A). Furthermore, we reproduced the risk stratification, my means of OS and DSS, in accordance with published data (Supplementary Figure 4B-4C) [6]. Concerning the Lund cohort, the unstable subtype covered a broad overlap of the predicted infiltrated (p53-like) and luminal subtype, whereas the squamous cell carcinoma were exclusively clustered to the predicted basal subtype (Supplementary Figure 3).

The p53 –like gene signature could be dichotomized into late cell cycle genes upregulated exclusively in the basal and luminal subtype (*AURKA/B*, *CCNB1/A2*, *MAD2L1*) and early cell cycle genes (*MDM4*, *CDK6*, *CDKN1A*), which may belong to the genuine p53-like downstream signature. In this study, no subtype specific enrichment of *TP53* pathway genes could be identified (Supplementary Figure 5A, 5D). Mainly genes involved in inflammation and immune infiltration (*FAS*, *NCAM1*, *CCL2*, *CD14*) remained in the so-called ‘p53-like’ subtype of the reduced gene panel. Gene set enrichment analyses showed an enrichment of inflammatory and immune-infiltration genes in the Chungbuk (FDR=7%, p=0.04) and the MDA cohort (FDR<25%, p=0.29) by using an independent immune signature from the Gene Ontology Database (Supplementary Figure 4C, 4D). Therefore, we suggest renaming this subtype as ‘infiltrated’. Given the excellent prognosis of these patients throughout cohorts, this seemed logic from a biological and clinical point of view. Curated luminal and basal breast cancer signatures [18, 19] showed a significiant enrichment in their respective subtypes of the MDA and Chungbuk cohorts has been validated by GSEA (Supplementary Figure 5, p<0.03). CD44 is known to cluster to the basal subtype, given its overexpression in squamous carcinoma (Supplementary Figure 8) [6, 20]. In our study however, we focused on urothelial carcinoma, which led to an assignment to the infiltrated subtype in each cohort. In fact, CD44 is also known for its involvement in inflammation and in leukocyte migration and homing [21, 22].

We further compared the clustering with our reduced geneset with the recently proposed MIBC subtypes of the TCGA cohort [12]. Again, all genes of the reduced geneset stably clustered to their respective subtypes (Supplementary Figure 7). The basal subtype covered the basal squamous TCGA subtype. The luminal infiltrated TCGA subtype was overlapping with the infiltrated subtype and finally the luminal and luminal papillary TCGA subtype was concordant to the luminal subtype.

### Outcome prediction and risk stratification

The identified MIBC subtypes were predictive for patient survival in the Mannheim cohort, despite its modest sample size. The luminal subtype showed worst outcome with an 8-year OS and DSS of 11% (p=0.028) and 17% (p=0.011). The infiltrated subtype showed best outcome with an 8-year OS of 46% and DSS of 73% (Figure 1B-1C). In order to investigate the impact of clinicopathologic characteristics and molecular subtypes on patient survival, we performed Cox's proportional hazards regression analysis (Figure 1D). Multivariate analyses were adjusted for the covariates with significance in univariate analyses: T2 vs. T3-T4, N+ vs. N0, AC vs. no AC and luminal vs. not-luminal. The only significant covariate remaining in the Cox regression model was the distinction between luminal vs. not-luminal tumors yielding a five-fold higher risk of disease specific death (HR=4.94, 95% CI: 1.56-15.63, p=0.007) in patients with luminal tumors, when treated with cystectomy only (Figure 1D and Table 2).

**Table 2:** Results of Cox proportional hazard analysis of independent risk factors for survival prediction

Cox regression analysis		DSS	
**Mannheim**	**HR**	**95% CI**	**p**
Luminal	4.94	1.56-15.63	0.007
**Chungbuk**	**HR**	**95% CI**	**p**
Age	1.06	1.01-1.10	0.009
pN+	4.31	1.72-10.81	0.002
Luminal	3.76	1.50-9.46	0.005
T2 vs. T3, T4	2.90	1.17-7.19	0.022
**MDA**	**HR**	**95% CI**	**p**
NAC	3.57	1.30-9.80	0.013

HR = hazard ration, CI = confidence interval, NAC = neoadjuvant chemotherapy.

This same tendency could be confirmed in the Chungbuk cohort. This cohort included less advanced tumors and a high amount of patients treated with adjuvant cisplatin chemotherapy. Comparing luminal and not-luminal patients, the poor outcome of luminal tumors could be confirmed (p=0.051) (Figure 2A-2D). In a Cox's regression model (adjusted for gender, TNM, age and luminal vs. not-luminal) the luminal subtype was associated with a higher risk of death (HR=3.76, 95% CI 1.50-9.46, p=0.005), however, positive lymph nodes were a stronger predictor (HR=4.31, 95% CI: 1.72-10.81, p=0.002). Interestingly adjuvant chemotherapy showed no impact on outcome (Table 2). The MDA cohort showed a significant stratification of DSS and OS under the condition that squamous carcinomas were included. However, squamous carcinoma were known to show inferior survival and to cluster exclusively to the basal subtype, as could be confirmed by our data (Supplementary Figure 4A-4C). Due to the exclusion of squamous carcinoma together with the strong impact of NAC (p=0.013, 95% CI 1.30-9.80) in multivariate analyses (adjusted for gender, NAC, cM, pT2 vs. pT3-4) no difference in outcome could be observed (Figure 3A-3D: p>0.03, Table 2).

### Subtype specific expression of drug targets

Drug target genes were not included in the subclassification gene set, in order to allow an independent investigation of their subtype attribution. *ERBB2* was exclusively expressed in the luminal subtype (p<0.003, Figure 4A). Immunohistochemistry confirmed its subtype specific expression and thus underlines its potential translational benefit by means of a targetable biomarker in luminal MIBC patients (Figure 4B).

The progesterone receptor (*PR*) and estrogen receptor 1 (*ESR1*) showed no subtype specific expression (data not shown). In contrast, androgen receptor (*AR*) and *ESR2* were significantly suppressed in the basal subtype (p=0.001 and p=0.046 respectively, Figure 4A). The *FGFR* gene family was also differentially expressed between subtypes, but the distribution varied between its family members *FGFR1*, *FGFR2* and *FGFR3*. The tyrosine kinases *FGFR1* and *PDGFRB* are exclusively expressed in the infiltrated subtype (p<0.003), whereas *FGFR3* was significantly enriched in the luminal subtype (Figure 4A). As many parallels with breast cancer became apparent, we further investigated the expression of relevant members of the claudin gene family. In concordance with breast cancer subtypes, the basal MIBC subtype showed a claudin-low molecular phenotype (p≤0.03, Supplementary Figure 6). *EGFR*, *ERBB4* and *FGFR4* showed no subtype specific expression (data not shown).

## DISCUSSION

Several independent studies revealed distinct molecular MIBC subtypes with different clinicopathological features and potential actionable drug targets [6–8, 11]. Given the heterogeneity of molecular bladder cancer phenotypes, robust and sensitive methods were requested for subtype validation [15–17]. Ideally, these methods should also be transferrable into clinical routine (e.g. Prosigna^®^, FDA approved) [23]. In the Mannheim cohort, the NanoString nCounter subtype screening, as a sensitive absolute quantification method, identified three distinct molecular subtypes with significantly different outcome, based on a reduced consensus panel of 36 genes. It is of note that our cohort included no squamous carcinoma and was exclusively treated with radical cystectomy in order to analyze the genuine course of MIBC subtypes.

The basal subtype was mainly characterized by the presence of cytokeratins (e.g. KRT14, KRT5). This subtype showed poor hormone receptor expression and low claudin expression, likewise the triple negative basal or claudin-low breast cancer subtype [24]. Basal and luminal MIBC showed an activation of late cell cycles genes (e.g. *AURKA*, *AURKB*) which seem to be strong discriminating factors toward the infiltrated subtype throughout the different cohorts. Thus, the suppression or dysfunction of early cell cycle genes as seen in the luminal and basal subtype may be deleterious for MIBC survival in accordance with recent data [25].

The so called ‘p53-like’ subtype has been shown to display mesenchymal and immune infiltration characteristics [9, 14]. Given its higher survival rates and the enriched immune signature (Figure 4C-4D), we renamed this subtype as ‘infiltrated’, as first referred to by Sjödhal et al. [7]. The infiltrated subtype showed best prognosis throughout the cohorts, nevertheless studies have shown that this subtype is resistant to NAC [6, 10]. Actually, the high expression of early cell cycle genes may suggest a functioning G1 checkpoint, thus supporting the idea of a more quiescent phenotype. Yet gene expression in the infiltrated subtype is difficult to interpret, as the expression may be diluted or enhanced by leukocyte infiltration. In this context, the assistance of immunohistochemistry may provide significant complementary information [9]. In accordance to recent data, the proportion of this subtype varies between cohorts indicating different degrees of infiltration [9]. This may explain in some extend the differing proportion of infiltrated tumors, showing indeed a favorable outcome.

Our luminal gene signature was characterized by an upregulation of surface proteins UPK2 and KRT20, in concordance with previous array studies. PPARG1, known to be a phenotype determining factor, was exclusively expressed in the luminal subtype. The same important role had been attributed to GATA3, a transcription factor upregulated in the luminal subtype [26]. However, this gene was not retained by the Venn diagrams for the consensus gene signature. CD24 is a luminal marker which, after reduction to the consensus geneset, changed to the basal subtype signature only in the Mannheim cohort (Figure 1). Given the uniqueness of this event throughout our validation cohorts, this may be ascribed to the low patient number of the Mannheim cohort.

The luminal phenotype seemed to be a more targetable phenotype considering for example the exclusive overexpression of *ERBB2*, *FGFR2-3* and hormone receptors. As these markers were not included in the clustering gene set, we delivered a strong confirmation for their subtype specificity. These findings urge prospective randomized trials with a subtype specific exposure with e.g. trastuzumab, tyrosine kinase inhibitors and hormone receptor antagonists.

The genuine disease course of chemotherapy-naïve MIBC without squamous carcinoma revealed that not the basal but the luminal subtype may present worst prognosis. In the MDA cohort, the squamous cell carcinoma, known to show poor survival, clustered to the basal subtype in accordance to published data [6]. When the later were excluded, multivariate analyses showed a significant impact of NAC on MIBC patient survival, making it impossible to make a judgment about the genuine disease course and the discrepancies of subtype specific survival between both studies. Interestingly, AC did not have such an influence on survival in the Chungbuk cohort [27]. The later did also confirm the poor prognosis of luminal MIBC, including only transitional cell carcinoma. The common nomenclature in breast cancer may suggest a poorer survival for basal carcinoma in contrast to luminal tumors. However, seen the exclusive high expression of *ERBB2* in the luminal subtype, outcome may rather be compared to HER2+ breast cancer, shown to present a significant inferior outcome compared to the basal subtype [28]. Survival analysis of the class 2 tumors from Hedegaard et al., alike our luminal subtype, confirmed its poor prognosis in non-muscle-invasive bladder cancer in contrast to the basal subtype [29]. The discrepancies of subtype specific survival between studies are mainly based on different treatment modalities and variant histology (squamous versus transitional cell carcinoma). Thus, consensus criteria are needed for future clinical trials for the sake of reproducibility. Given the limited number of patients enrolled in this study, further prospective validation on larger cohorts is needed.

Previous studies found MIBC to be mainly dichotomized into two subcategories, either by the predominant expression of genes attributed to a luminal or basal phenotype or the expression of distinct and opposing pathways [8, 30, 31]. Both can be found in the present analysis, the latter especially by the expression of early and late cell cycle genes. Both concepts harmonize when genes condense into three clusters.

## MATERIALS AND METHODS

### Tumor cohorts

Formalin-fixed paraffin-embedded tissue (FFPE) samples of muscle-invasive urothelial carcinoma were collected after radical cystectomy and bilateral lymphadenectomy from chemotherapy-naïve patients (n=47) of the University Medical Center Mannheim. All patients gave informed consent and the retrospective analysis was approved by the relevant institutional review board. All specimens have been reviewed by an experienced uropathologist (AH) according to the TNM classification of 2010 (UICC). *In silico* validation was performed on the MDA (n=58, GSE48276), Chungbuk (n=61, GSE13507) and Lund (n=51, GSE32894) cohort [6, 7, 32]. Squamous cell carcinomas were excluded and exclusively patients with muscle invasive transitional cell carcinomas after radical cystectomy were kept for analysis.

### Gene expression profiling

Total RNA was extracted from 10μm FFPE PCR sections of tumor samples of the Mannheim cohort with a bead-based system (XTRACT kit, STRATIFYER Molecular Pathology GmbH, Cologne, Germany). Matched hematoxylin and eosin stained sections were performed for the selection of samples containing a minimum amount of 30% tumor tissue. RNA quality has been assessed by Nanodrop and qRT-PCR. An amount of 100ng total RNA was analyzed by the nCounter standard chemistry. The curated gene panel included 64 biomarkers based on enrichment analyses of recent literature and consensus data [6, 8, 14, 15]. (Supplementary Figure 1) Potential drug targets were further tested for their subtype specific expression and thus were not included in the subtyping panel.

The nCounter assay was normalized using the geometric mean of 6 reference genes (*CALM2*, *RPL37A*, *B2M*, *TUBB*, *GAPDH* and *G6PD*) and 6 internal positive controls. Negative background subtraction was performed by 8 negative internal controls. The nSolver software 2.5 was used for data preprocessing.

The *in silico* datasets were downloaded as processed data from the Gene Expression Omnibus and cBioportal database. All raw intensities were log2 transformed and quantile normalized. Clustering was performed on the preselected genes (n=64) also included in the nCounter panel. The top three gene clusters were assigned in semi-supervised manner to groups of basal, luminal and p53-like genes. The overlay of the respective groups was analyzed by Venn diagrams among the four cohorts. Using this reduced consensus gene panel, the patients were clustered in three groups by unsupervised hierarchical clustering with Pearson correlation as similarity measure and Ward as agglomeration method.

### Immunohistochemistry

Immunohistochemical stainings were carried out on a BenchMark Ultra (Ventana, Tucson, Arizona) using clinical-grade (CLIA) antibodies according to the manufacturer's instructions. The antibodies were diluted as follows: CK5 1:50 (Diagnostic BioSystems), CK20 1:50 (Dako) and Her2/neu 1:1000 (Dako). All stainings were reviewed by two pathologists (ME, AH).

### Statistical methods

Clinicopathologic characteristics were compared using Fisher's exact test for categorical and the Kruskal-Wallis test for continuous variables. Overall survival (OS) and disease specific survival (DSS) were analyzed by the Kaplan-Meier method and tested for significance by the log-rank test. Univariate and multivariate analysis were performed by a Cox proportional hazards regression model with a forward selection method. For this purpose, we dichotomized the subtype covariate into ‘luminal’ and ‘non-luminal’ MIBC. P-values <0.05 were judged as significant. Statistics were conducted using R version 3.3.1., Graph Pad Prism v7 and SPSS v20.0. Gene set enrichment analyses were conducted using the GSEA software v2.2.3 (Broad Institute). Gene signatures were downloaded from the MSigDB.

## SUPPLEMENTARY MATERIALS FIGURES AND TABLES


