# Supplementary material for: Subclassification, survival prediction and drug target analyses of chemotherapy-naïve muscle-invasive bladder cancer with a molecular screening
Source: Oncotarget. 2018 May 25;9(40):25935–45. doi: 10.18632/oncotarget.25407 (PMC5995241; doi:10.18632/oncotarget.25407)
Supplement: Supplementary file 1 [file oncotarget-09-25935-s001.pdf]

Subclassification, survival prediction and drug target analyses of chemotherapy-naïve muscle-invasive bladder cancer with a molecular screening

SUPPLEMENTARY MATERIALS

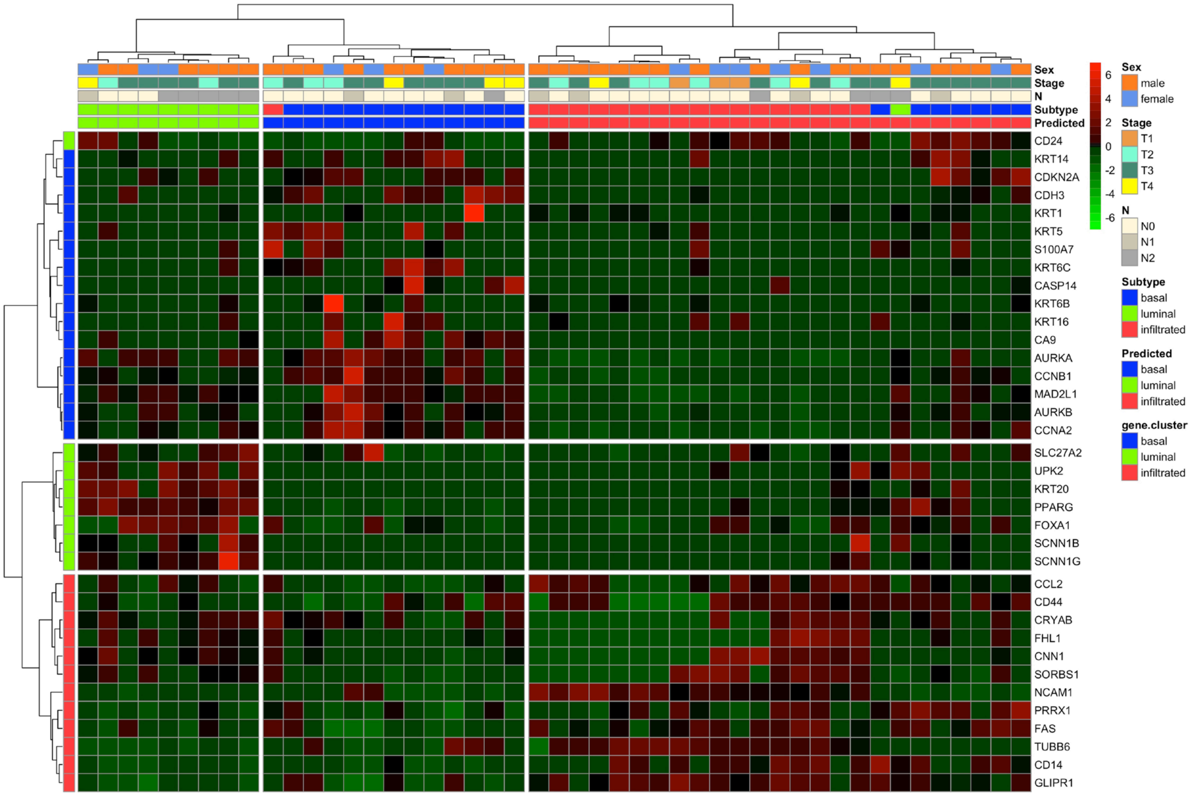

Supplementary Figure 1: Initial nCounter gene panel including 64 biomarkers, known to be enriched in the luminal, basal and p53-like (here termed as infiltrated) MIBC subtypes.

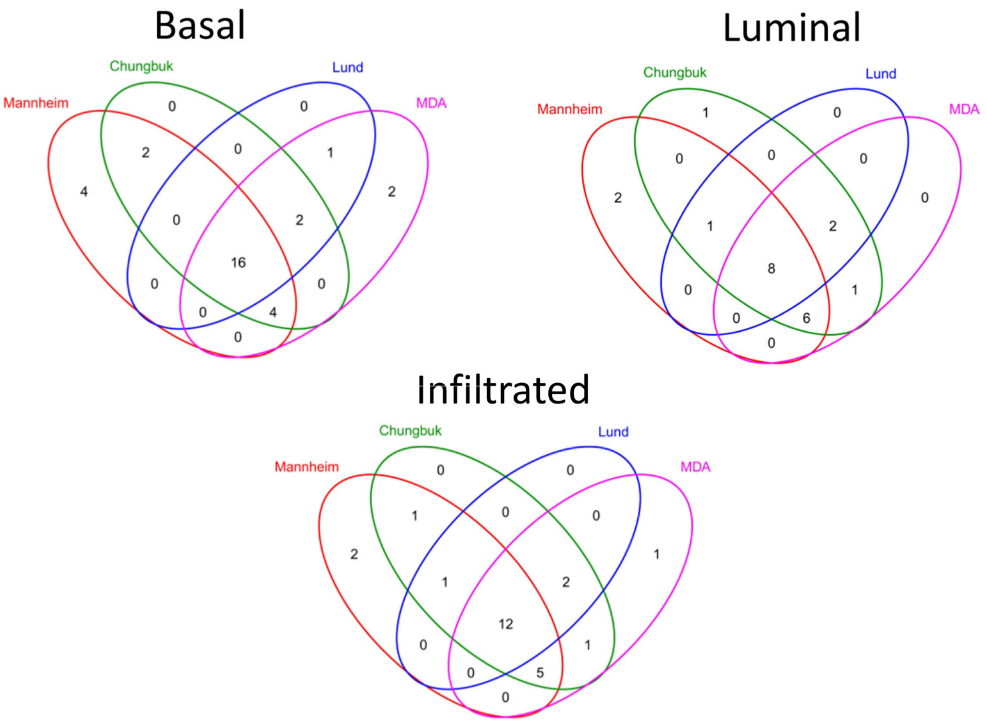

Supplementary Figure 2: The overlay of the basal, luminal and p53-like subtypes from the Mannheim, Lund, MDA and Chungbuk cohort resulted in a reduced consensus gene panel of 36 stable clustering genes.

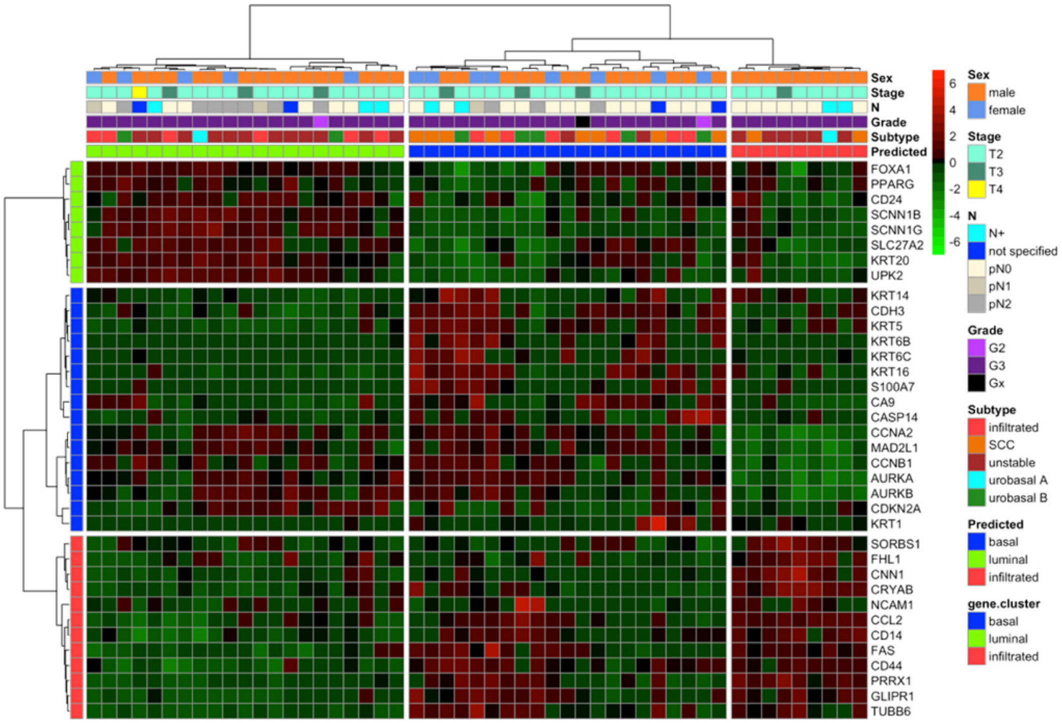

Supplementary Figure 3: MIBC subtype classification of the Lund cohort by gene expression profiling of the reduced consensus geneset based on in silico microarray data (n=51, GSE32894). Survival analyses have not been performed as no detailed treatment, histology and reason of death data were available.

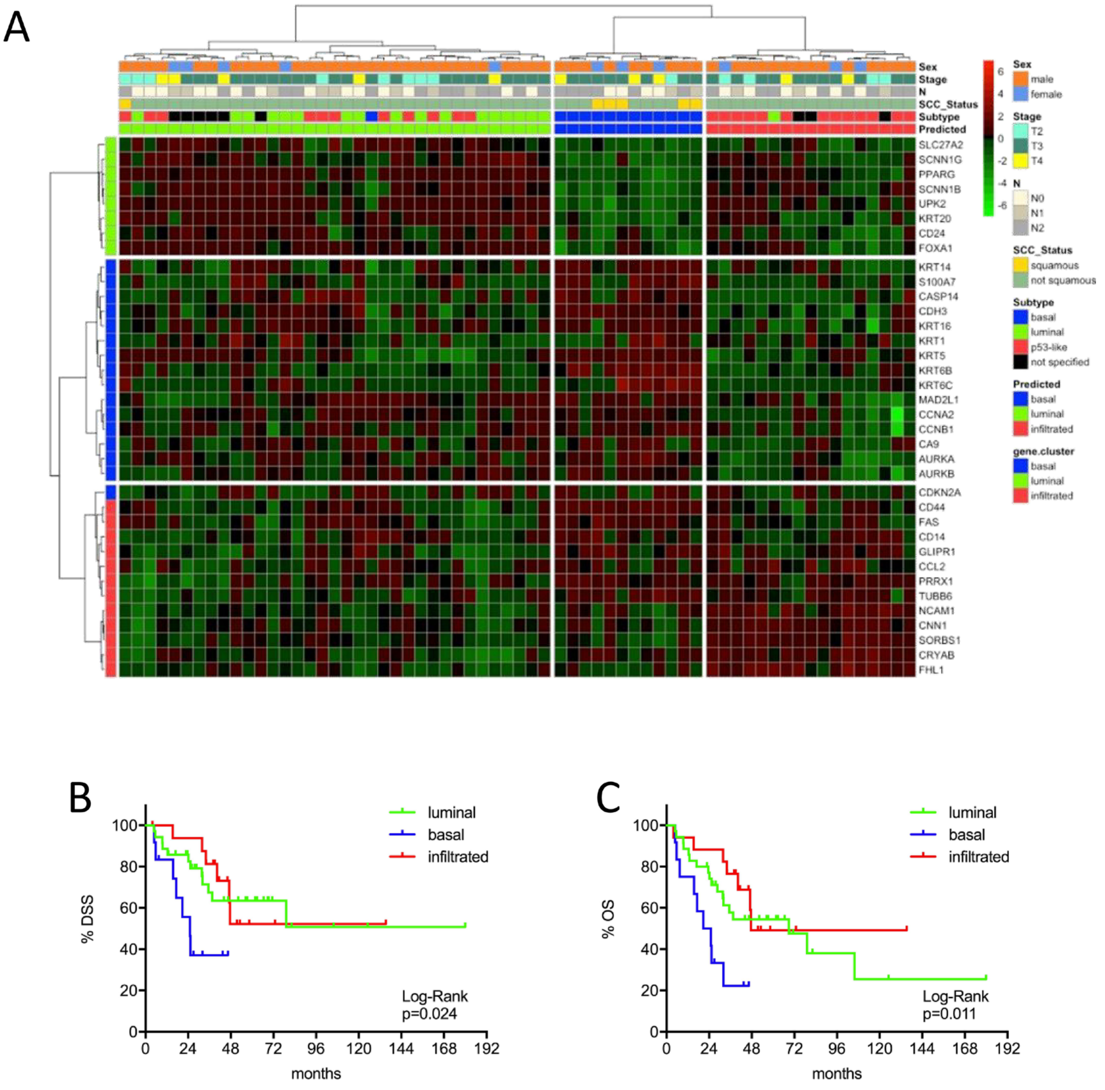

Supplementary Figure 4: (A) MIBC subtype classification of the MDA cohort (n=64) including squamous cell carcinoma, by gene expression profiling of the reduced consensus geneset based on in silico microarray data (GSE48276). **(B, C)** Kaplan-Meier plots of overall survival (OS) and disease specific survival (DSS) of the basal, luminal and infiltrated subtype.

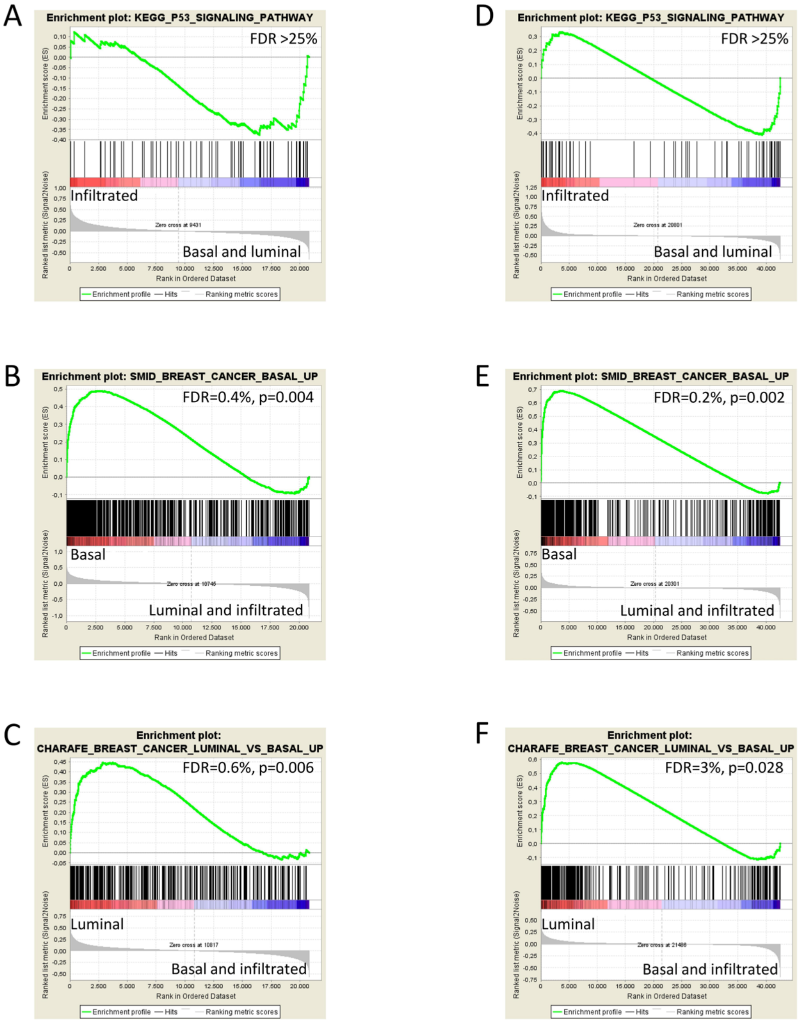

Supplementary Figure 5: Gene set enrichment analysis of the TP53 pathway from KEGG database and curated basal and luminal gene signatures publically available on MsigDB (MDA: A, B, C, Chungbuk:D, E, F). In both cohorts, the TP53 gene signature showed no enrichment **(A, D)**. The MDA cohort **(B, C)** and the Chungbuck cohort **(E, F)** showed a highly significant enrichment of basal and luminal biomarkers in their respective basal and luminal subtypes defined by the reduced gene panel.

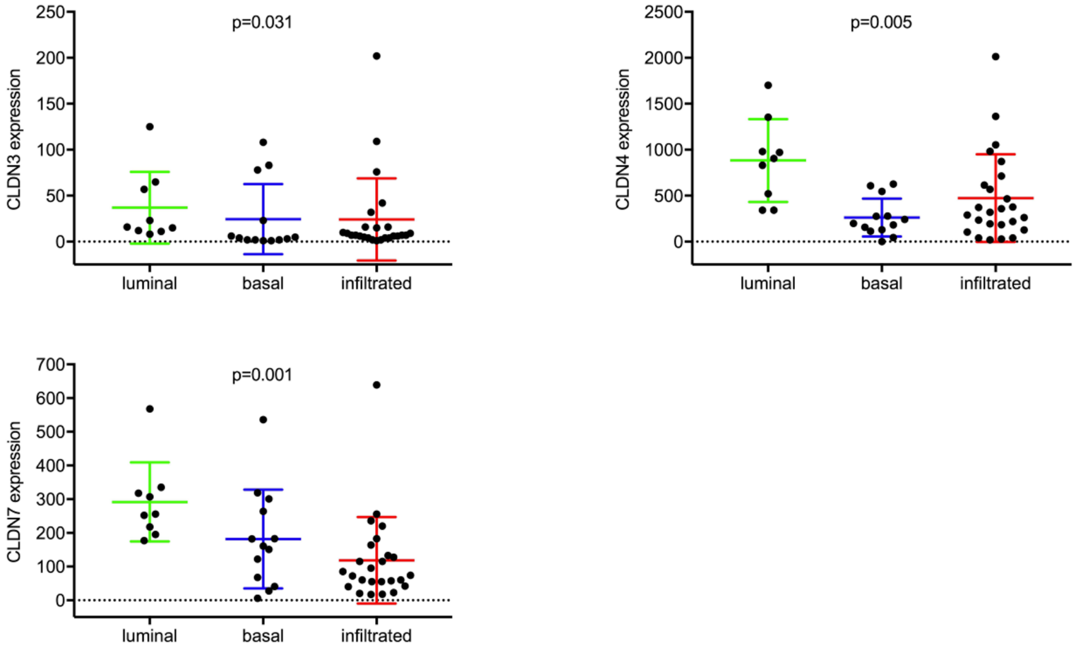

Supplementary Figure 6: Claudin 3, 4 and 7 were tested for their subtype specific expression. Absolute quantification of transcript levels was based on normalized nCounter counts. The basal and infiltrated subtypes showed a claudin-low molecular phenotype.

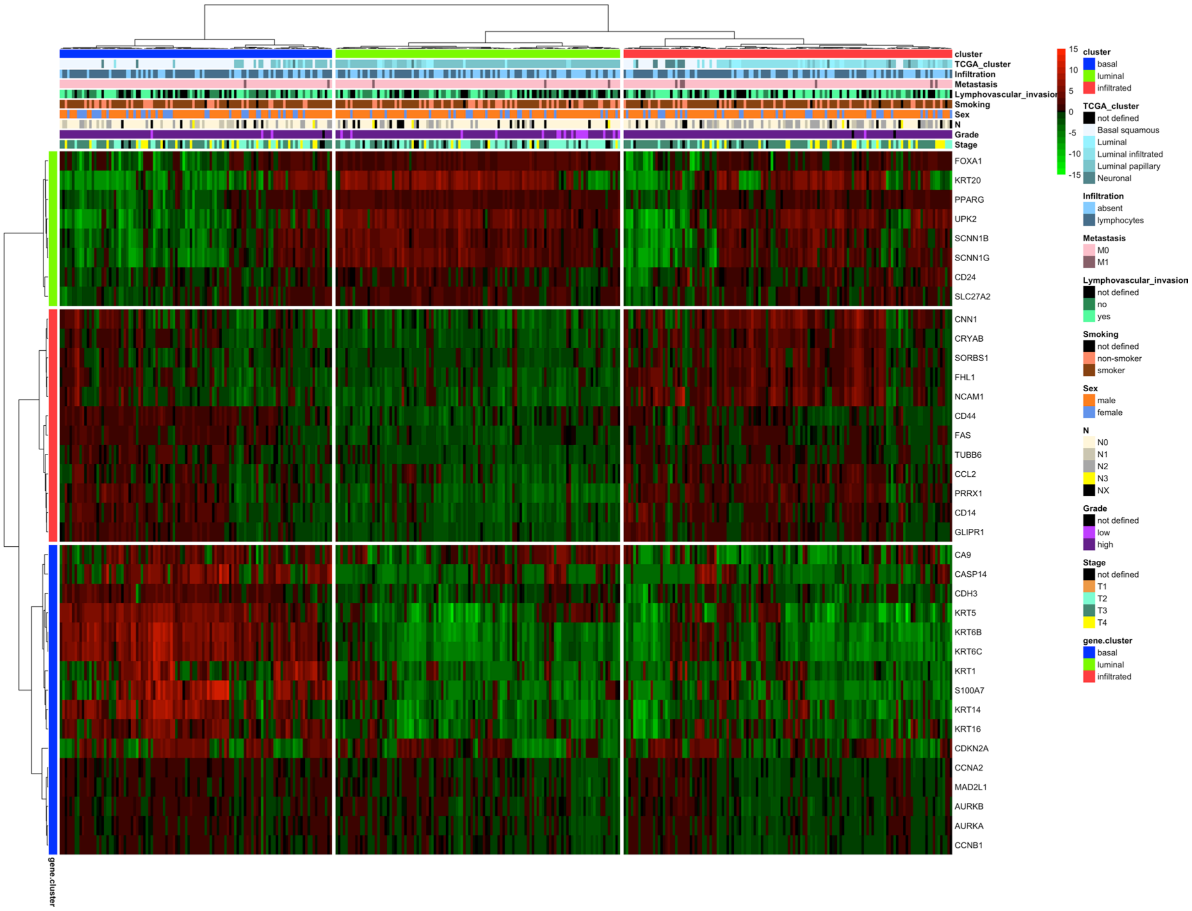

Supplementary Figure 7: MIBC subtype classification of the TCGA cohort by gene expression profiling of the reduced consensus geneset based on in silico RNA-Seq data (n=364, cBioportal). Exclusively transitional cell carcinoma are assessed. Survival analyses have not been performed as no detailed treatment and reason of death data were available.

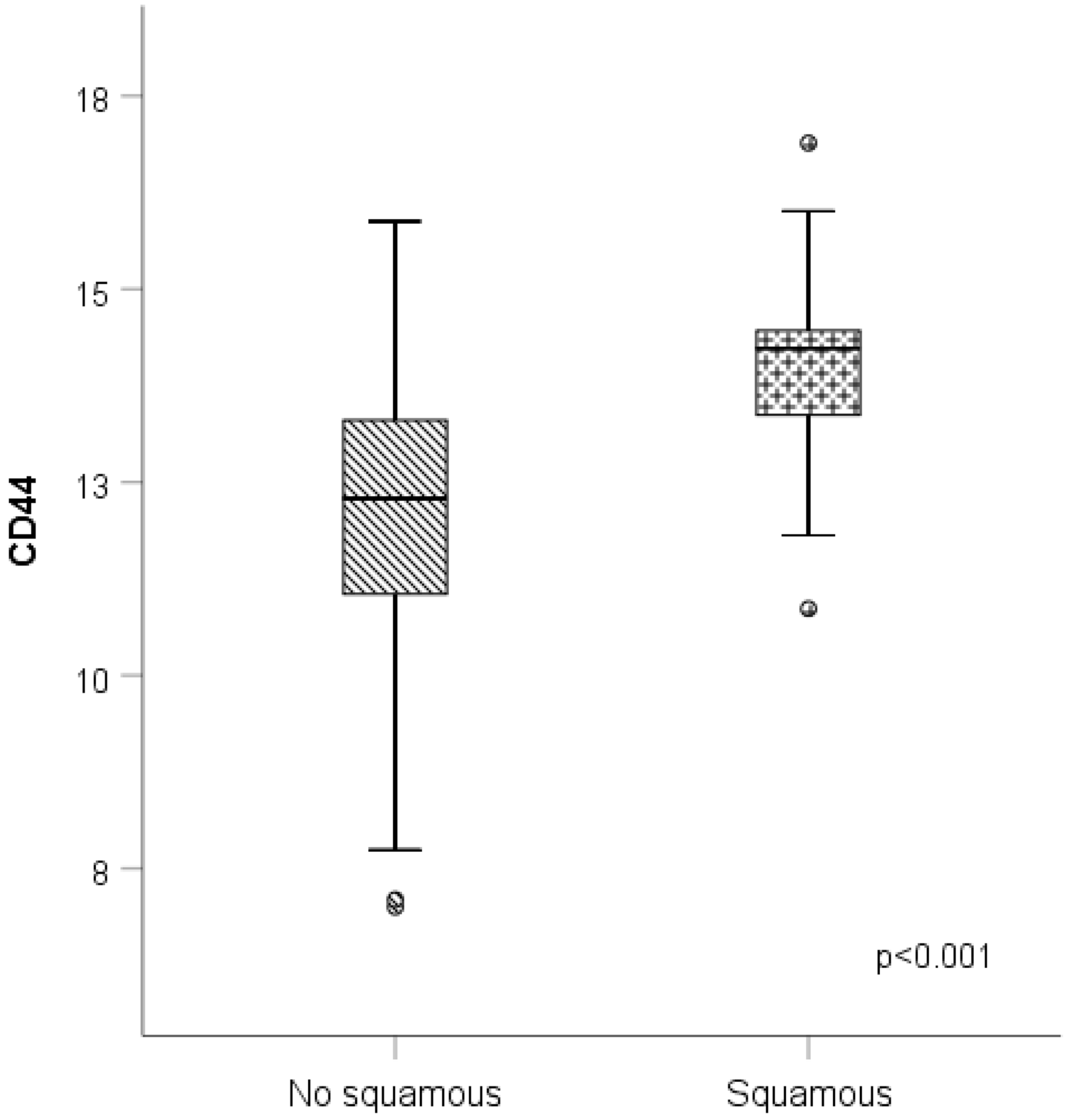

Supplementary Figure 8: Differential expression of CD44 between squamous (n=45) and non-squamous (n=364) carcinoma of the TCGA cohort (p<0. 001).

Supplementary Table 1: Clinicopathologic characteristics of the MDA cohort

Cohort characteristics	Total	(%)	Luminal	(%)	Basal	(%)	Infiltrated	(%)	p-value
Cohort size	58		31	(53)	12	(21)	15	(26)	
Median age	68		63		70		69		0.305
Female	9	(16)	5	(16)	1	(8)	3	(20)	0.801
Male	49	(85)	26	(84)	11	(92)	12	(80)	
**TNM Stage**									
pT2	13	(22)	6	(19)	2	(17)	5	(33)	0.463
pT3	35	(60)	21	(68)	6	(50)	8	(53)	
pT4	10	(17)	4	(13)	4	(33)	2	(13)	
pN+	36	(62)	20	(65)	7	(58)	9	(60)	0.938
cM+	2	(3)	2	(7)	0	(0)	0	(0)	1.000
**Additional Therapy**									
NAC (n=39)	9	(23)	6	(26)	0	(0)	3	(43)	0.097
**Histology**									
Urothelial	46	(79)	23	(74)	10	(83)	13	(87)	0.629
Other	12	(21)	8	(26)	2	(17)	2	(13)	

NAC = neoadjuvant chemotherapy.

Supplementary Table 2: Clinicopathologic characteristics of the Chungbuk cohort

Cohort characteristics	Total	(%)	Luminal	(%)	Basal	(%)	Infiltrated	(%)	p-value
Cohort size	61		31	(51)	13	(21)	16	(28)	
Median age	66		67		72		62		0,036
Female	13	(21)	8	(26)	4	(31)	1	(6)	0,141
Male	48	(79)	23	(74)	9	(69)	16	(94)	
**TNM Stage**									
pT2	31	(51)	14	(45)	5	(39)	12	(71)	0,025
pT3	19	(31)	8	(26)	8	(62)	3	(18)	
pT4	11	(18)	9	(29)	0	(0)	2	(12)	
pN+ (n=60)	14	(23)	6	(19)	5	(39)	3	(19)	0,433
cM+	6	(10)	4	(13)	0	(0)	2	(12)	0,535
**Additional Therapy**									
AC	26	(43)	15	(48)	4	(31)	7	(41)	0.553

AC = adjuvant chemotherapy.
